# In Situ Formation of Silver Nanoparticles Induced by Cl-Doped Carbon Quantum Dots for Enhanced Separation and Antibacterial Performance of Nanofiltration Membrane

**DOI:** 10.3390/membranes13080693

**Published:** 2023-07-25

**Authors:** Yi-Fang Mi, Jia-Li Liu, Wen Xia, Shu-Heng He, Bao-Qing Shentu

**Affiliations:** 1 State Key Lab of Chemical Engineering, College of Chemical and Biological Engineering, Zhejiang University, 38 Zheda Road, Hangzhou 310027, China; 2 Key Laboratory of Advanced Textile Materials and Manufacturing Technology and Engineering Research Center for Eco-Dyeing & Finishing of Textiles, Ministry of Education, Zhejiang Sci-Tech University, Hangzhou 310018, China; 3 Zhejiang Shenlan New Material Technology Co., Ltd., Jiandei 311606, China

**Keywords:** nanofiltration, antibacterial performance, silver nanoparticles, carbon quantum dot, visible light irradiation

## Abstract

Polyamide (PA) nanofiltration (NF) membranes suffer from biofouling, which will deteriorate their separation performance. In this study, we proposed a strategy to incorporate silver nanoparticles (Ag NPs) into PA NF membranes in situ, in order to simultaneously enhance water permeability and antibacterial performance. The chloride-doped carbon quantum dots (Cl-CQDs) with photocatalytic performance were pre-embedded in the PA selective layer. Under visible light irradiation, the photogenerated charge carriers generated by Cl-CQDs rapidly transported to silver ions (Ag^+^ ions), resulting in the in situ formation of Ag NPs. The proposed strategy avoided the problem of aggregating Ag NPs, and the amount of Ag NPs on the membrane surfaces could be easily tuned by changing silver nitrate (AgNO_3_) concentrations and immersion times. These uniformly dispersed Ag NPs increased membrane hydrophilicity. Thus, the obtained thin film nanocomposite Ag NPs (TFN-Ag) membrane exhibited an improved water flux (31.74 L m^−2^ h^−1^), which was ~2.98 times that of the pristine PA membrane; meanwhile, the sodium sulfate (Na_2_SO_4_) rejection rate was 96.11%. The sterilization rates of the TFN-Ag membrane against Escherichia coli (*E. coli*) and Staphylococcus aureus (*S. aureus*) were 99.55% and 99.52%, respectively. Thus, this facile strategy simultaneously improved the permeability and antibacterial property of PA NF membranes.

## 1. Introduction

Due to the boom in population growth and the development of industry, the demand for water purification technology requires sustainable growth [1]. NF membranes, with their merits of energy efficiency, environmental benefits, and low cost, have been widely applied in many areas, including seawater desalination [2], wastewater treatment [3], water softening [4], and so on. Improving the water flux and selectivity of NF membranes is an ongoing objective for NF membrane design. In addition, most PA NF membranes suffer from inevitable biofouling, which is one of the key problems to be solved in membrane separation processes [5]. Membrane biofouling results from microbial growth and biofilm formation. Disinfectants such as chlorine have been used for pre-treatment; however, bacterial proliferation still exists because the surviving bacteria can migrate to the membrane surface. Furthermore, harmful disinfection byproducts may be formed when disinfectants are used, and some disinfectants themselves result in the degradation of PA thin film composite (TFC) membranes [6,7]. Apart from addressing the issue of decreasing membrane separation performance caused by biofouling, the growing pursuit of pathogen-free water is another reason for developing antimicrobial membranes [8]. Therefore, simultaneously improving the separation performance and antibacterial properties of NF membranes is highly desirable. 

To enhance the separation performance and antibacterial ability of PA TFC membranes, many strategies, such as topological or chemical surface engineering and incorporating some hydrophilic and/or antimicrobial nanomaterials [9,10,11,12], have been proposed. Of these approaches, the incorporation of nanomaterials, which can provide enhanced free volume in the PA selective layer and additional nano-pores/channels for water transport, has received great significant interest [12,13,14]. Meanwhile, a variety of nanomaterials, including Ag NPs [15,16,17], zinc oxide nanoparticles [18], copper nanoparticles [19], titanium dioxide nanoparticles [20], graphene oxide nanosheets [21,22], and curcumin-enriched sodium dodecyl benzenesulfonate (Cur-NaDBS) nanoparticles [23], have been successfully applied to effectively inhibit bacterial proliferation on membranes. Currently, the incorporation of these dual-functional nanomaterials to the PA selective layer is mainly through ex situ or in situ methods, forming thin film nanocomposite (TFN) membranes. The addition of pre-made nanomaterials is referred to as the ex situ method [24]. With this strategy, PA selective layers produce non-selective defects due to functional nanomaterials’ mediocre dispersibility and compatibility. In addition, the weak interaction between the PA membrane and these ex situ incorporated nanomaterials results in the leakage of functional nanomaterials. These phenomena lead to serious deterioration of membrane structure and separation performance during continued operation [25,26,27]. Therefore, the in situ method, where the nanomaterials are generated and incorporated into the PA matrix simultaneously, has been proposed to address the above-mentioned limitations. Compared to the ex situ method, the nanomaterials generated by the in situ method show better dispersity and compatibility with the PA matrix. In addition, no extra procedure is needed for the separation and purification of the nanomaterials, making this method much easier than the ex situ method. Therefore, a paradigm shift is highly desired for the in situ integration of Ag NPs to achieve TFN membranes with excellent separation performance and antibacterial ability.

Carbon quantum dots (CQDs) have attracted broad research interest in membrane fabrication, due to their small particle sizes and abundant functional groups [28,29,30]. Numerous studies demonstrated an increase in water permeability by incorporating CQDs as a nanofiller into the PA selective layer [31,32]. However, it is still difficult to simultaneously enhance the water permeability and antibacterial abilities of TFN membranes. Recently, the doping of Cl into CQDs was observed to facilitate an enhanced separation of photoexcited charge carriers, and enable the rapid conversion of Ag^+^ ions to metallic Ag NPs under visible light irradiation [33]. In this study, we proposed a strategic in situ formation of Ag NPs induced by Cl-CQDs under visible light irradiation to endow the PA TFC membranes with high water permeability and good antibacterial performance. In this research, Cl-CQDs acted as anchor sites that were previously embedded in pristine PA TFC membranes that were obtained through interfacial polymerization reactions between piperazine (PIP) and trimesoyl chloride (TMC). Then, Ag NPs were formed in situ on the membrane surface by immersing the as-prepared membrane in AgNO_3_ aqueous solution under visible irradiation. The consequent formation of Ag NPs was generated by the embedded Cl-CQDs that accelerated the photogenerated charge carriers to the Ag^+^ ions [33], contributing to the formation of Ag NPs. The successful in situ incorporation of Ag NPs onto the membrane was confirmed. The presence of Ag NPs on the membrane surface properties, separation performance, and antibacterial ability were systemically analyzed. These results shed light on the preparation of advanced NF membranes with high water permeability and antibacterial ability through a facile and applicable method.

## 2. Experiment

### 2.1. Materials

The Cl-CQDs were supplied by XFNANO Materials Tech Co., Ltd (Nanjing, China).; polysulfone (Psf) ultrafiltration membranes were obtained from Zhongke Ruiyang Membrane Technology Co., Ltd. (Beijing, China); PIP, TMC, n-hexane, AgNO_3_, and inorganic salts, including sodium chloride (NaCl), Na_2_SO_4_, magnesium sulfate (MgSO_4_), and nitric acid (HNO_3_), were all supplied by Macklin Biochemical (Shanghai) Co., Ltd.; *S. aureus* (KCTC 3881), *E. coli* (ATCC 47076), and the corresponding culture mediums were purchased from Luwei Biological Technology Co., Ltd.(Zibo, Shandong, China). Home-made deionized water was utilized in all of the experiments.

### 2.2. Preparation of TFN-Ag Membranes

**Figure 1:**
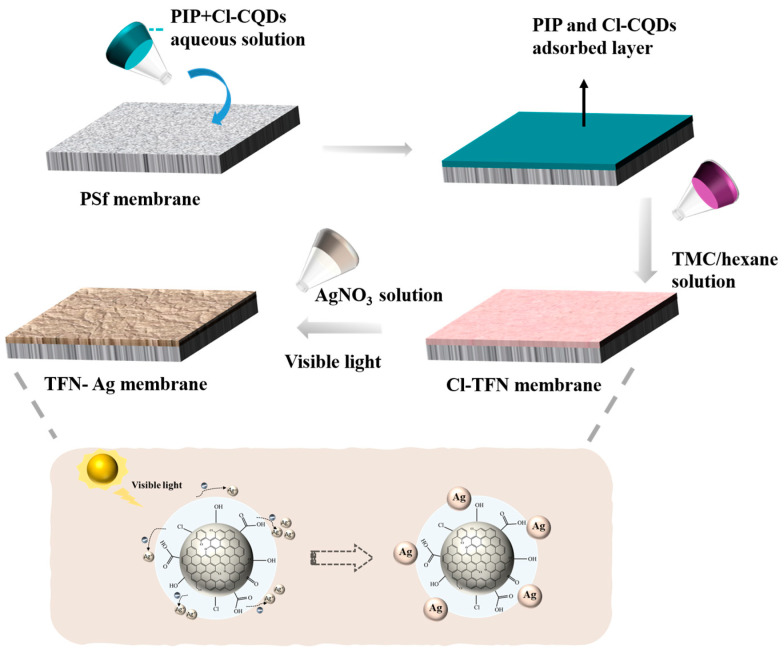
Schematic of strategic preparation of TFN-Ag membranes through the in situ formation of Ag NPs induced by Cl-CQDs under visible light irradiation.

The manufacturing processes of the TFN-Ag membranes are schematically presented in Figure 1. A volume of 20 mL of PIP (1 wt%) aqueous solution containing Cl-CQDs (0.05 mg mL^−1^) was poured onto the Psf ultrafiltration membranes. After 2 min, the aqueous solution was drained from the Psf membrane surface. When no obvious droplets on the surface of Psf membrane were observed, the TMC/n-hexane solution was poured onto the Psf membrane surface for 1 min. The nascent PA membrane was generated through interfacial polymerization between PIP and TMC. Then, the membranes were soaked in AgNO_3_ aqueous solutions with different concentrations for a certain time (1–15 min) under visible light irradiation, followed by oven drying at 50 °C for 15 min, in order to obtain the TFN-Ag membranes. A xenon lamp (PLS-SXE-300 W) was adopted for the generation of visible light using a 420 nm filter to cut off ultraviolet light, and the temperature of the AgNO_3_ solution was controlled by circulating condensate water. Specifically, when the immersion time of the AgNO_3_ solution was 3 min, the obtained NF membranes were abbreviated as TFN-Ag 1, TFN-Ag 2, TFN-Ag 4, TFN-Ag 6, and TFN-Ag 8, according to the concentration of AgNO_3_ solution (1, 2, 4, 6, and 8 mg mL^−1^, respectively). 

For contrast studies, the pristine PA membrane was prepared through the interfacial polymerization between PIP and TMC, and abbreviated as TFC membrane, while the PA membrane containing Cl-CQDs was abbreviated as Cl-TFN membrane.

### 2.3. Characterizations

The Ag NPs formed in situ by Cl-CQDs were confirmed with an ultraviolet-visible (UV-vis) spectrometer (UV-2600). The morphology and relative size of Ag NPs formed in the Cl-CQDs solution were visualized via scanning electron microscopy (SEM, GeminiSEM500, Jena, Germany).

The membrane surface morphologies were evaluated with SEM, and the existence of Ag elements was confirmed by energy dispersive X-ray (EDX) spectra. The membrane chemical structures were characterized using attenuated total reflectance Fourier transform infrared spectroscopy (ATR-FTIR, Nicolet Aratar 370). The surface chemical composition was further determined via X-ray photoelectron spectroscopy (XPS, Thermo Fisher Scientific ESCALAB Xi+). The membrane hydrophilicity was confirmed using a video contact angle system (DSA-20, Germany) with the sessile drop method. 

### 2.4. Separation Performances of TFN-Ag Membranes

The NF performance of the membranes was investigated with a lab-made cross-flow membrane filtration apparatus at 25 °C. Before the test, all of the tested membranes with an effective surface area of 22.4 cm^2^ were pre-pressurized at 0.6 MPa for at least 30 min until the water flux became stable. The salt solution (NaCl, Na_2_SO_4_, and MgSO_4_, 1 g L^−1^) was tested. The flux (J, L m^−2^ h^−1^) and rejection (R, %) were calculated with Equations (1) and (2), respectively:

(1) $$J=\frac{V_{p}}{At}$$

(2) $$R=(1-\frac{C_{p}}{C_{f}})\times 100\%$$

where V_p_ is the permeated water volume (L), A is the effective surface area of membrane (m^2^), t is the filtration time (h), and C_p_ and C_f_ are the solute concentrations of the permeate and feed solution, respectively, obtained by a Mettler Toledo electrical conductivity meter (FE-30). The data presented are the averages of three parallel experiments. 

### 2.5. Characterizations of Membrane Antibacterial Performance

The antibacterial performances of the TFC, Cl-TFN, and TFN-Ag 4 membranes were assessed using *S. aureus* (Gram-positive) and *E. coli* (Gram-negative). The culture media, reagents, and utensils used in this experiment were all pre-sterilized with ultraviolet light for 1 h before the tests. The bacteria, i.e., *S. aureus* or *E. coli*, were cultivated at 37 °C in Luria–Bertani (LB) broth, and suspensions containing 10^8^ colony forming units per mL (CFU mL^−1^) of bacteria were obtained. A 100 μL, 10^7^ CFU mL^−1^ suspension was inoculated on the LB solid medium; then, the TFC, Cl-TFN, and TFN-Ag 4 membranes were placed faced-down to contact with the LB agar. The inhibition zone was observed after culturing at a constant temperature of 37 °C for 24 h. 

A volume of 100 μL of diluted bacterial solution (10^7^ CFU mL^−1^) was dropped onto the sterilized membrane surface, following incubation at 37 °C for 3 h. Then, 100 μL of diluted solution (diluted 1 × 10^6^ times) was coated on the solid LB medium, and cultured at 37 °C for 12 h. The bacteriostasis rate (*BR*) of the membranes was obtained by counting the viable bacterial colonies and using the following equation:

(3) $$BR=\left(1-\frac{N_{m}}{N_{b}}\right)\times 100\%$$

where *N_b_* and *N_m_* are the numbers of bacterial colonies on the control group and TFN-Ag membrane, respectively. The data presented are the averages of three parallel experiments.

### 2.6. Silver Release Experiments

The release rates of the Ag^+^ ions from the TFN-Ag 4 membrane were assessed via batch experiments. The membrane sample (2 × 2 cm^2^) was immersed in 20 mL of DI water and placed on an orbital shaker (SYC-2012, Crystal) under 100 rpm at room temperature. Water was collected, and an equal amount of DI water was added every 24 h. The concentrations of released Ag^+^ ions from the TFN-Ag 4 membrane in the collected water were quantitatively determined using an inductively coupled plasma mass spectrometer (ICP-MS, iCAP6300, PerkinElmer, Waltham, MA, USA). To dissolve the Ag on the membrane surface completely, the TFN-Ag 4 membrane was digested by 1 wt% HNO_3_ aqueous solution. Subsequently, the obtained solution was determined via ICP-MS to acquire the total amount of Ag on the TFN-Ag 4 membrane. 

## 3. Results and Discussion

### 3.1. The In Situ Formation of Ag NPs Induced by Cl-CQDs

**Figure 2:**
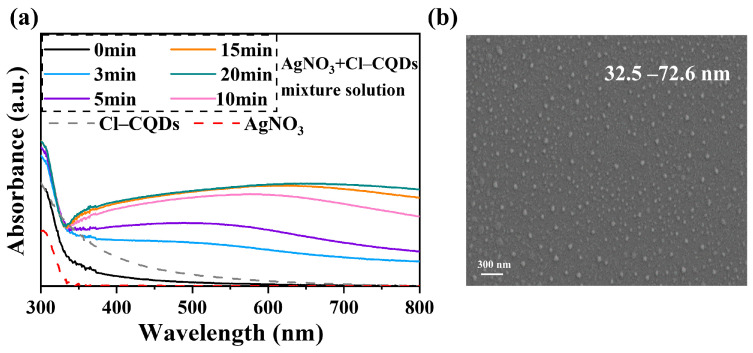
UV–visible spectra of AgNO_3_ solution, Cl-CQDs solution, and AgNO_3_ solution containing Cl-CQDs under visible light irradiation for different times (**a**); SEM images of formed Cl-CQDs/Ag NPs nanocomposites (**b**).

To confirm the Ag NPs formation induced by Cl-CQDs under visible light irradiation, 0.05 mg mL^−1^ commercial Cl-CQDs were added into the AgNO_3_ solution, and the variations in the formation process were monitored using UV–vis spectra. As shown in Figure 2a, the UV–visible spectra of the AgNO_3_ and Cl-CQDs mixture solution were completely different from that of the single solution. Under visible light, strong adsorption originating from the surface plasmon resonance of the generated Ag NPs was found in the UV–vis spectra [34], confirming the successful in situ generation of Ag NPs induced by Cl-CQDs. The intensity of the adsorption peak increased with increasing irradiation time within 15 min, while further prolonging the irradiation time resulted in no apparent changes. The increased adsorption peak demonstrated the continuous formation of Ag NPs. Moreover, the Cl-CQDs yielded a rapid reduction process, and the in-situ formation of Ag NPs was accomplished within 15 min. The generated Cl-CQDs/Ag NPs showed a broad size distribution, i.e., 32–72 nm (Figure 2b), which was consistent with the broad adsorption peak in Figure 2a. Cl-doped CQDs have been demonstrated to generate an additional energy level, which would be beneficial for enhancing the photocatalytic activity of Cl-CQDs triggered by visible light. This behavior would accelerate the transportation of photogenerated charge carriers towards Ag^+^ ions, contributing to the rapid formation of Ag NPs [33]. Therefore, we concluded that the Cl-CQDs induced the in situ formation of Ag NPs under visible light irradiation and generated Cl-CQDs/Ag NPs nanocomposites. These results made it possible for Cl-CQDs to induce Ag NPs formation on the PA membrane surface in situ.

### 3.2. Preparation of TFN-Ag Membranes via In Situ Formation Induced by Cl-CQDs

**Figure 3:**
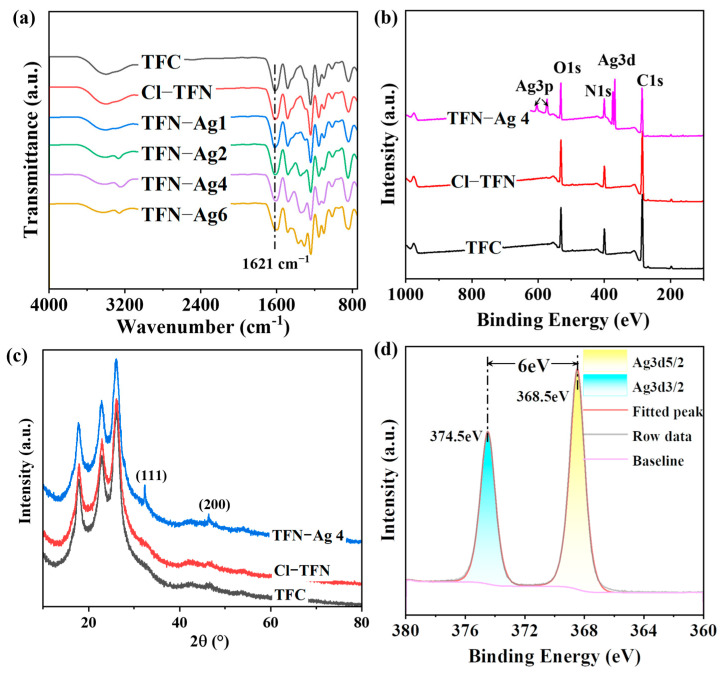
(**a**) ATR-FTIR spectra and (**b**) XPS spectra of TFC, Cl-TFN, and TFN-Ag membranes; (**c**) XRD spectra of TFC, Cl-TFN, and TFN-Ag 4 membranes; (**d**) high-resolution Ag3d XPS spectrum of TFN-Ag 4 membrane.

To realize in situ Ag NPs formation on the membrane surface, Cl-CQDs, which act as anchors, were introduced in the PA selective layer during interfacial polymerization. Then, by immersing the resultant membrane in AgNO_3_ solution under visible light irradiation, TFN-Ag (1–6) membranes with Ag NPs decorated on the surfaces were obtained. The chemical properties of the TFN-Ag membranes were confirmed with ATR-FTIR and XPS. The characteristic peak at 1621 cm^−1^ attributed to amide groups was observed in all of the characterized membranes, demonstrating the successful generation of PA selective layers [35]. XPS analysis provided a method to quantitatively analyze the chemical composition of the membrane surfaces. The characteristic peaks of C, N, and O elements at 285, 400, and 531 eV, respectively, were revealed in the XPS spectra of the TFC, Cl-TFN, and TFN-Ag (1–6) membranes (Figure 3b); these were consistent with the chemical compositions of the PA selective layers. No Ag elements were detected in the TFC and Cl-TFN membranes. After immersion in AgNO_3_ solution, Ag elements were detected (Table 1) and apparent Ag3d signals could be detected on the surface of the TFN-Ag 4 membrane. Moreover, new peaks at 38.1° and 44.3° appeared in the XRD spectrum for TFN-Ag 4, which were ascribed to the (111) and (200) diffractions of the face-centered cubic structure of Ag NPs [36] (Figure 3c). In addition, the high-resolution Ag3d XPS spectrum was analyzed in detail (Figure 3d) to further confirm the status of Ag that existed on the TFN-Ag 4 membrane surface. Doublet signals were observed at 368.5 eV and 374.5 eV, corresponding to Ag 3d_5/2_ and Ag3d_3/2_ peaks, respectively. Moreover, the spin energy difference of these two peaks was equal to 6.0 eV, indicating the generation of metallic Ag NPs [37]. The Ag3p signals were attributed to the existence of silver chloride (AgCl) caused by the Cl-CQDs, and the resultant AgCl would also be beneficial for improving antibacterial performance [38,39]. The stronger intensity of the Ag3d signals than those of the Ag3p signals manifested the dominant role of Ag NPs. Furthermore, the elemental atomic contents are presented in Table 1; TFN-Ag 4 possessed a higher O/N ratio (1.059) than that of the TFC membrane (0.997), indicating the relatively lower cross-linking degree of TFN-Ag 4 [40]. Collectively, the TFN-Ag (1–6) membrane where Ag NPs were formed in situ on the surface induced by Cl-CQDs under visible light irradiation was successfully manufactured.

**Figure 4:**
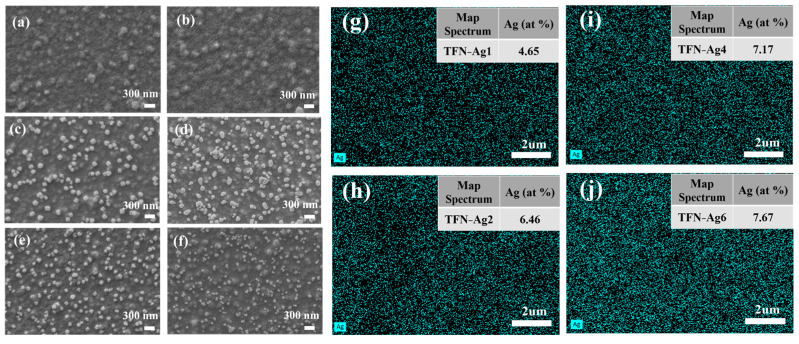
Surface morphologies of (**a**) TFC, (**b**) Cl-TFN, and TFN-Ag membranes with different AgNO_3_ concentrations: (**c**) 1 mg mL^−1^, (**d**) 2 mg mL^−1^, (**e**) 4 mg mL^−1^, and (**f**) 6 mg mL^−1^ (immersion time was 3 min).

Furthermore, the surface morphologies of the TFN-Ag (1–6) membranes were characterized using SEM. As depicted in Figure 4, the TFC membrane possessed a typical nodular surface morphology of the PA membrane, indicating that successful interfacial polymerization reactions happened [41]. No obvious morphological changes in the Cl-TFN membrane were observed after the incorporation of Cl-CQDs. In contrast, Ag NPs were observed on the TFN-Ag (1–6) membranes. The Ag atomic content on the membrane surface increased from 4.65 to 7.67% with increasing the AgNO_3_ concentration from 1 to 6 mg mL^−1^, as evidenced by the EDX results (Figure 4g–j). This in situ method for the formation of Ag NPs triggered by Cl-CQDs could effectively tune the surface morphologies, and even the separation performance of the TFN-Ag membranes (discussed later), by regulating the loaded content of Ag NPs.

**Figure 5:**
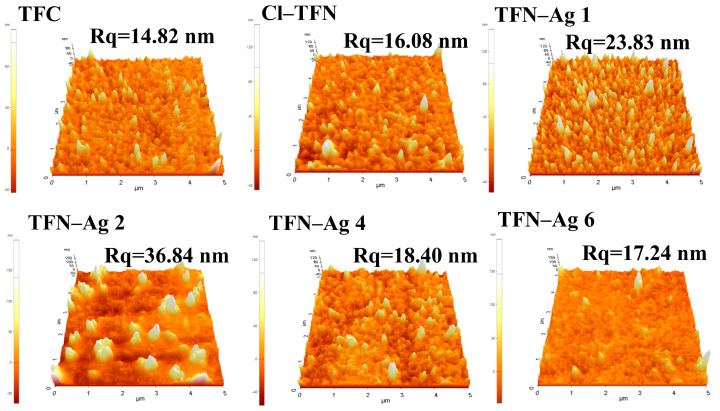
AFM images of TFC, Cl-TFN, and TFN-Ag membranes.

The surface roughness of the TFC, Cl-TFN, and TFN-Ag membranes are shown in Figure 5. Due to the small particle size (1.5–5.5 nm) and good water dispersity of the Cl-CQDs, no significant difference in the surface morphology was observed in the TFC and Cl-TFN membranes, and these two membranes showed similar surface roughness. With the in situ growth of Ag NPs, the surface roughness initially increased to 36.84 nm, and then increased to 17.24 nm when the AgNO_3_ concentration was increased from 1 to 6 mg mL^−1^. As shown in Figure 4, large Ag NPs were observed on the TFN-Ag 1 and TFN-Ag 2 membranes, resulting in a significant increase in membrane roughness. When further increasing the AgNO_3_ concentration, smaller and even-distributed Ag NPs were generated, leading to decreased surface roughness. The rearrangement of PA chains when immersed in the AgNO_3_ solution would decrease the membrane surface roughness, while more Ag NPs would enhance the membrane roughness [42,43]. The combined effect of these two reasons may be responsible for the changes in membrane roughness of the TFN-Ag membranes.

**Figure 6:**
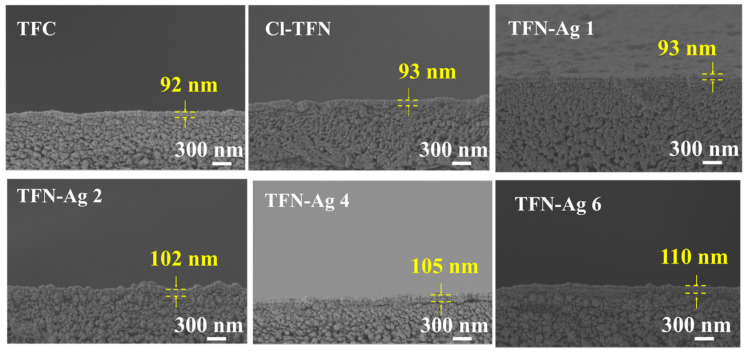
Cross-sectional morphologies of TFC, Cl-TFN, and TFN-Ag (1–6) membranes.

**Figure 7:**
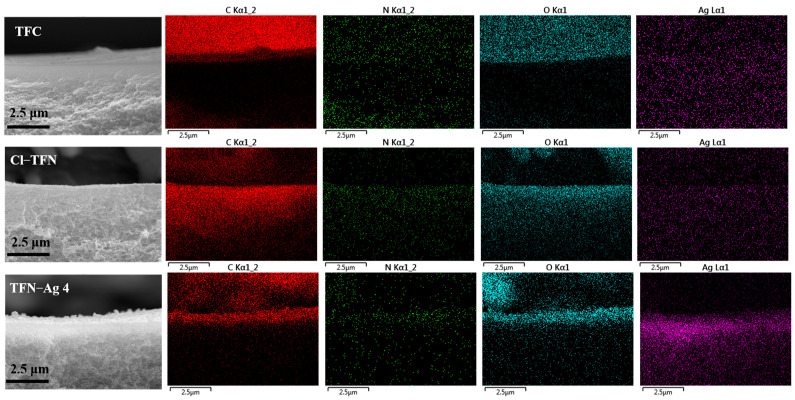
EDX mapping spectra of cross sections of TFC, Cl-TFN, and TFN-Ag 4 membranes.

Figure 6 shows the cross-sectional SEM images of the TFC, Cl-TFN, and TFN-Ag membranes. The thickness of all of the characterized membranes was in a range of 95–110 nm. The membrane thickness had no obvious change, indicating that the in situ generation of Ag NPs did not affect the PA structure in the selective layer. The EDX spectra of cross sections of the TFC, Cl-TFN, and TFN-Ag 4 membranes are presented in Figure 7. Due to the spraying of gold before the SEM characterizations, small amounts of Ag elements were detected in the TFC and Cl-TFN membranes. By contrast, the Ag contents in the TFN-Ag 4 selective layer were obviously higher than those in the cross sections of the TFC and Cl-TFN membranes. This result suggests that Ag NPs existed in the TFN-Ag 4 membranes. All of the results demonstrated the successful preparation of TFN-Ag membranes through the in situ formation of Ag NPs induced by Cl-CQDs under visible light irradiation.

### 3.3. Separation Performance of Membranes

**Figure 8:**
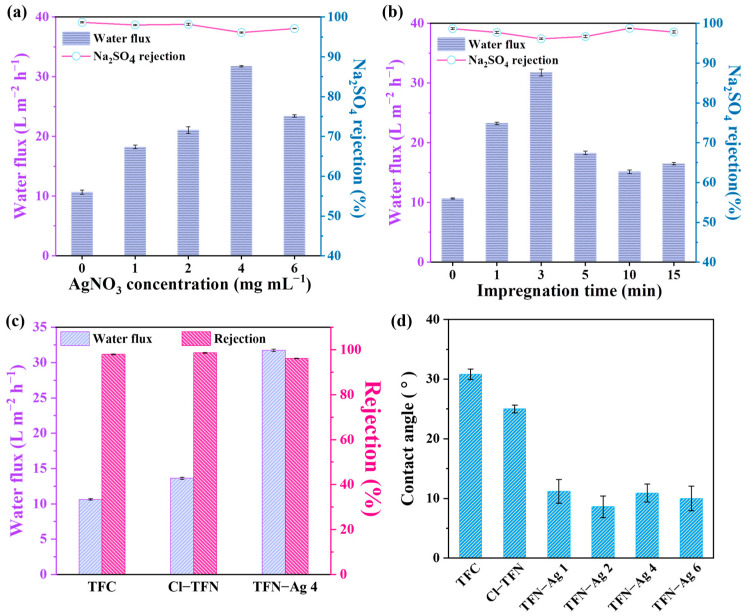
Nanofiltration performances of TFN-Ag membranes prepared by various (**a**) AgNO_3_ concentrations and (**b**) impregnation times. (**c**) Comparisons of TFN-Ag 4 separation performance with TFC and Cl-TFN membranes. (**d**) Water contact angles of TFC, Cl-TFN, and TFN-Ag membranes.

The AgNO_3_ concentration and impregnation time were considered to be two important factors that influenced formation of the Ag NPs and the resulting membrane. Hence, as shown in Figure 8, the separation performances of the TFN-Ag membranes were systematically investigated by varying the fabrication conditions, e.g., the impregnation times and the AgNO_3_ concentrations. After being immersed in AgNO_3_ solution, all of the characterized TFN-Ag membranes exhibited higher water flux than those of the TFC and Cl-TFN membranes. Specifically, it was observed that the water flux significantly improved from 10.68 to 31.74 L m^−2^ h^−1^ as the concentrations of AgNO_3_ were increased from 0 to 4 mg mL^−1^. Upon further increasing the AgNO_3_ concentration, the water flux decreased to 23.42 L m^−2^ h^−1^. The increased water flux was mainly ascribed to the improved membrane hydrophilicity with the in situ incorporation of Ag NPs by Cl-CQDs (Figure 8d). However, higher AgNO_3_ concentrations resulted in higher amounts of Ag NPs on the membrane surface, which increased the transport resistance to water; therefore, decreased water flux was observed [44]. The slightly lower Na_2_SO_4_ rejection may be ascribed to the lower cross-linking degree, as the acyl chloride groups were hydrolyzed during the impregnation process. These hydrolyzed acyl chloride groups would not be crosslinked during heat treatment, leading to lower cross-linking degree of membrane. Furthermore, the impregnation time of the AgNO_3_ solution significantly influenced the separation performance of the TFN-Ag membranes. As shown in Figure 8b, the water flux first increased to 31.74 L m^−2^ h^−1^ when the impregnation time was 3 min. When the impregnation time was prolonged to 15 min, the water flux reduced to 16.47 L m^−2^ h^−1^. The Na_2_SO_4_ rejection rates changed from 96.11% to 98.76%, which was slightly lower than the Na_2_SO_4_ rejection rate of the TFC membrane (98.65%). Therefore, the optimized preparation condition was immersion in 4 mg mL^−1^ AgNO_3_ aqueous solution for 3 min; then, the optimal membrane possessed improved water flux and high Na_2_SO_4_ rejection.

The TFN-Ag 4 outperformed the TFC and Cl-TFN membranes, as shown in Figure 8c. The TFC membrane possessed a water flux of 10.64 L m^−2^ h^−1^ and a 98.6% Na_2_SO_4_ rejection rate. The incorporation of Cl-CQDs slightly increased the water flux to 13.63 L m^−2^ h^−1^, while maintaining the Na_2_SO_4_ rejection rate (98.6%). Unlike the Cl-TFN membrane, the water flux of the TFN-Ag 4 membrane enhanced to 31.74 L m^−2^ h^−1^, which was ~2.98 times as much as the TFC membrane; meanwhile, the Na_2_SO_4_ rejection rate was 96.11%. The generated Ag NPs were well distributed on the surface of the TFN-Ag membrane without aggregation (Figure 4), which did not enhance the transport resistance to water, but increased the membrane hydrophilicity (Figure 8d); both were conducive to enhancing the water flux of the membrane [45]. The decreased water contact angle of the Cl-TFN membrane was ascribed to the incorporation of hydrophilic Cl-CQDs. After the in situ generation of Ag NPs, the water contact angles of the TFN-Ag membranes markedly decreased. For hydrophilic membranes, the rougher surface is beneficial for decreased water contact angles [46]. However, the water contact angles of the TFN-Ag 4 and TFN-Ag 6 membranes were lower than that of the Cl-TFN membrane, which possessed similar surface roughness. This result indicates that the in situ generation of Ag NPs was conducive to enhancing membrane hydrophilicity. Thus, we concluded that rougher surfaces and the in situ generation of Ag NPs both contributed to the decreased water contact angles. Therefore, a TFN-Ag membrane with improved water permeability was obtained via the in situ formation of Ag NPs on the membrane surface induced by Cl-CQDs under visible light irradiation.

**Figure 9:**
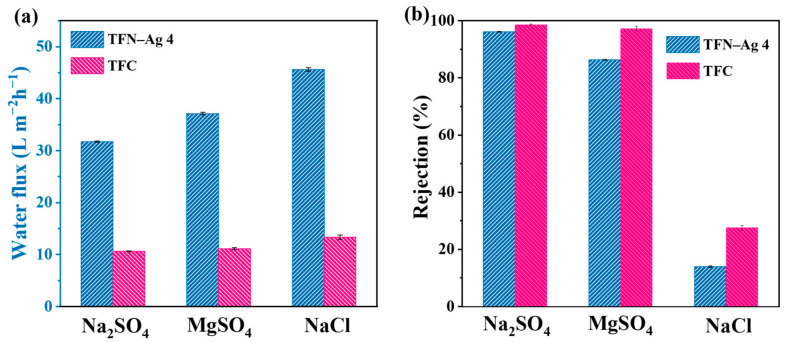
Separation performances of TFC and TFN-Ag 4 membranes: (**a**) water flux and (**b**) salt rejection rates.

In Figure 9, TFN-Ag 4 possessed a higher water flux than TFC when tested with four inorganic salts. The zeta potentials of the TFC and TFN-Ag 4 membranes were −14.58 and −21.09 mV at pH 7, respectively; thus, the salt rejections of the TFC and TFN-Ag 4 membranes both decreased in the following order: Na_2_SO_4_ > MgSO_4_ > NaCl, showing the rejection trend of negatively charged membranes [47,48]. There were high electrostatic repulsions between the negative membrane surface and sulfate ions, so the rejection rates of Na_2_SO_4_ and MgSO_4_ were higher than that of NaCl. The Mg^2+^ ions decreased the effective charge amount on the membrane surface, resulting in relatively low MgSO_4_ rejection. As can be seen in Figure 9b, the salt rejection rates of the TFN-Ag 4 membrane were all lower than those of the TFC membranes. This was because of the lower cross-linking degree of TFN-Ag 4, which had been proven by the XPS results (Table 1). The low cross-linking degree led to more acyl chloride groups hydrolyzing to carboxylic acid groups, resulting in a higher surface negative zeta potential. Therefore, we concluded that the separation performance of TFN-Ag 4 was determined by both steric hindrance and the Donnan effect.

### 3.4. Antibacterial Performance of TFN-Ag Membrane

**Figure 10:**
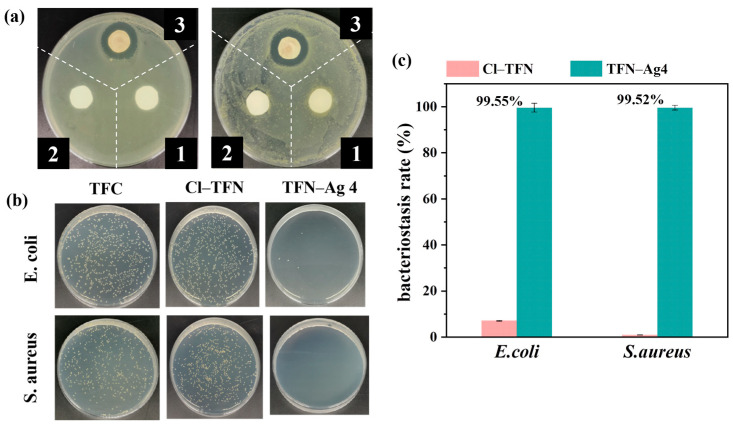
(**a**) Bacteriostatic zone experiment of TFC (1), Cl-TFN (2), and TFN-Ag 4 (3) membranes on *E. coli* (**left**) and *S. aureus* (**right**); plate count results of all the membranes including photographs of the bacterial culture plates (**b**); and the corresponding bacteriostasis rates (**c**). The control group denoted the bacteria culture in contact with the TFC membrane.

Good antibacterial properties of the membranes were also expected apart from their high separation performance, in order to meet the criteria of real applications. Thus, Gram-positive *S. aureus* and Gram-negative *E. coli* were used as model bacteria to ascertain the antibacterial performance of the TFC, Cl-TFN, and TFN-Ag 4 membranes. The results are shown in Figure 10. Compared with the TFC membrane, no obvious improvement to antibacterial activity of the Cl-TFN membrane was observed. However, the superior antibacterial performance of TFN-Ag 4 was proven by the inhibition zone test. Whether the TFN-Ag 4 membrane faced *E. coli* or *S. aureus*, apparent inhibition zones appeared around the TFN-Ag 4 membrane. In addition, the antibacterial activity of the membranes was also quantitatively assessed via the plate count method. Massive surviving *E. coli* and *S. aureus* bacterial colonies could be seen after being cultured with the TFC and Cl-TFN membranes, while almost no microbial colonies survived on the TFN-Ag 4 membrane. The TFN-Ag 4 membrane significantly reduced bacterial viability for both *S. aureus* and *E. coli*, further demonstrating its excellent antibacterial effect. Taking the TFC membrane as the control group, the bacteriostasis rates of TFN-Ag 4 against *E. coli* and *S. aureus* were 99.55 and 99.52%, respectively, which meant that the in situ formation of Ag NPs induced by Cl-CQDs on the membrane surface endowed the TFN-Ag 4 membrane with a strong antibacterial property. This was because the bacterial cell membranes were destroyed by the released Ag^+^ ions from the TFN-Ag 4 membrane, which in turn disrupted their cellular functions [49,50]. In addition, the Cl-CQD/Ag NPs nanocomposites could generate reactive oxygen species, which would contribute to bacteria inactivation [51].

**Figure 11:**
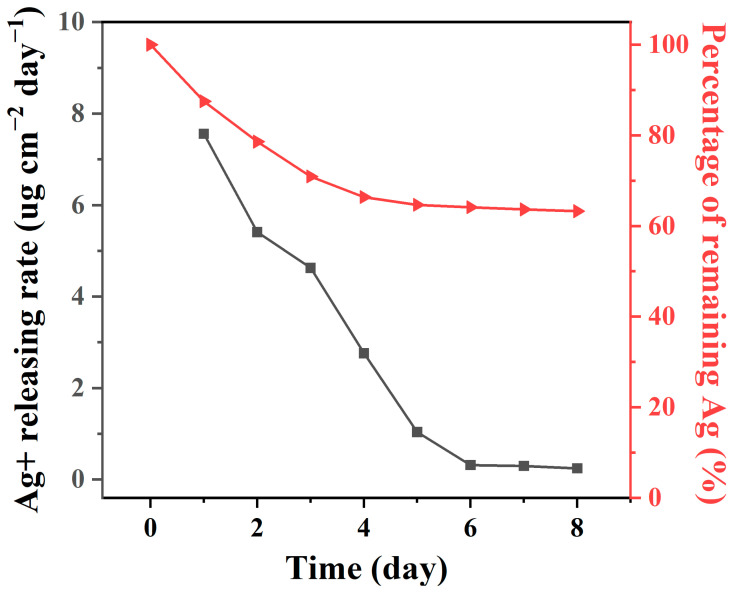
Releasing rate of Ag^+^ ions and the remaining percentage of Ag on the TFN-Ag 4 membrane.

To confirm and evaluate the duration of the antibacterial activity of the TFN-Ag 4 membranes, the released amount of the Ag^+^ ions was characterized via ICP–MS in an 8-day continuous examination. Figure 11 shows the Ag^+^ ions release rate and residual percentage of Ag on the TFN-Ag 4 membrane within 8 days. The release rate of the Ag^+^ ions was at a high level in the first 5 days, and then maintained a stable level (0.245 μg∙cm^−2^∙day^−1^). The prominently rapid Ag^+^ ion release rate in the initial stage resulted from the leakage of loosely bound Ag^+^ ions and the existence of AgCl. After 8 days, there was still 63.24% of Ag weight remaining on the TFN-Ag 4 membrane, while the weight loss rate of Ag on the TFN-Ag 4 membrane was estimated to be ~0.41%/day for the total amount of Ag. The total amount of Ag on the TFN-Ag 4 membrane was calculated to be 60.46 μg∙cm^−2^. The antibacterial ability of the TFN-Ag 4 membrane was estimated to last for 159 days, according to the Ag^+^ ion release rate, indicating the long-term antibacterial performance capability of the TFN-Ag 4 membrane. These results collectively demonstrate that the in situ generation of Ag NPs induced by Cl-CQDs enabled the robust incorporation of Ag NPs on the membrane, significantly improving the antibacterial performance.

## 4. Conclusions

In this study, a strategy for the in situ generation of Ag NPs induced by Cl-CQDs under visible light irradiation was proposed to construct PA TFN-Ag membranes. The embedded Cl-CQDs served as anchors to promote a rapid photogenerated charge carrier migration towards Ag^+^ ions, and induced Ag NPs to be uniformly distributed on the membrane surface. By optimizing the growth conditions, the surface properties of the membrane were regulated. The resultant TFN-Ag 4 membranes exhibited a high water flux of ~31.74 L m^−2^ h^−1^, which was ~2.98 times as much as the TFC membrane; meanwhile, the Na_2_SO_4_ rejection rate was 96.11%. The in situ formed Ag NPs endowed the as-prepared TFN-Ag 4 membrane with excellent antibacterial properties, and the sterilization rates against *E. coli* and *S. aureus* were 99.55% and 99.52%, respectively. The stronger antibacterial capability combined with higher permeability make TFN-Ag membranes potentially promising for actual wastewater treatment applications. In the future, we will modify the Cl-CQDs to enhance the interactions between Cl-CQDs and Ag NPs to realize a controlled release of Ag^+^ ions, and thus avoid health concerns. More types of nanoparticles, such as copper oxide, zinc oxide, etc., are expected to be used for in situ generation on the membrane surface with the aid of Cl-CQDs. Our strategy offers a paradigm shift toward the facile preparation of TFN membranes with improved permeability and antibacterial properties for diverse water treatment and solutes separation.

## Figures and Tables

**Table 1:** The elemental composition analyzed by XPS measurements.

Membrane	Surface Elemental Composition (at%)				
	C	N	O	Cl	Ag
TFC	70.19	14.48	14.44	0.88	0
Cl-TFN	69.89	12.84	16.38	0.89	0
TFN-Ag 4	62.53	14.22	15.07	1.56	6.9

## Data Availability

Not applicable.

## Abbreviations

Name	Abbreviation
nanofiltration	NF
polyamide	PA
thin film composite	TFC
thin film nanocomposite	TFN
silver nanoparticles	Ag NPs
silver nitrate	AgNO_3_
chloride-doped carbon quantum dots	Cl-CQDs
carbon quantum dots	CQDs
*Escherichia coli*	*E. coli*
*Staphylococcus aureus*	*S. aureus*
piperazine	PIP
trimesoyl chloride	TMC
sodium chloride	NaCl
sodium sulfate	Na_2_SO_4_
magnesium sulfate	MgSO_4_
ultraviolet–visible spectrometer	UV–vis spectrometer
scanning electron microscopy	SEM
energy dispersive X-ray spectra	EDX spectra
attenuated total reflectance Fourier transform infrared spectroscopy	ATR-FTIR
X-ray photoelectron spectroscopy	XPS
Luria–Bertani broth	LB broth
colony forming units per mL	CFU mL^−1^
